# Improvement of recovery yield of macro-organismal environmental DNA from seawater samples

**DOI:** 10.1007/s44211-023-00280-1

**Published:** 2023-02-09

**Authors:** Qianqian Wu, Toshifumi Minamoto

**Affiliations:** Graduate School of Human Development and Environment, Kobe University, 3-11 Tsurukabuto, Nada-Ku, Kobe City, Hyogo 657-8501 Japan

**Keywords:** Buffer-ATL, Environmental DNA (eDNA), Marine sample, MiFish, RNAlater, Specific amplification

## Abstract

In recent years, environmental DNA (eDNA) technology has been used in a variety of water environments. Environmental DNA concentrations in marine samples tend to be lower than those in freshwater samples, and few studies have explored methods to improve the recovery yields of eDNA from seawater samples. In this study, we compared different seawater preservation solutions (RNAlater or ATL) to improve eDNA yields. The eDNA concentrations of vertebrate and invertebrate species were compared using species-specific eDNA assays, and the number of detected fish and their compositions were compared using metabarcoding analysis. ATL treatment resulted in significantly higher eDNA yields for both vertebrate and invertebrate species than RNAlater treatment. Metabarcoding analysis revealed non-significant effects of preservation on the number of detected species and species composition. These results suggest that ATL treatment improves DNA yields without changing the species composition compared with the commonly used RNAlater treatment. The findings of this study will reduce false-negative outcomes and provide highly reliable results in future biological surveys.

**Graphical abstract:**

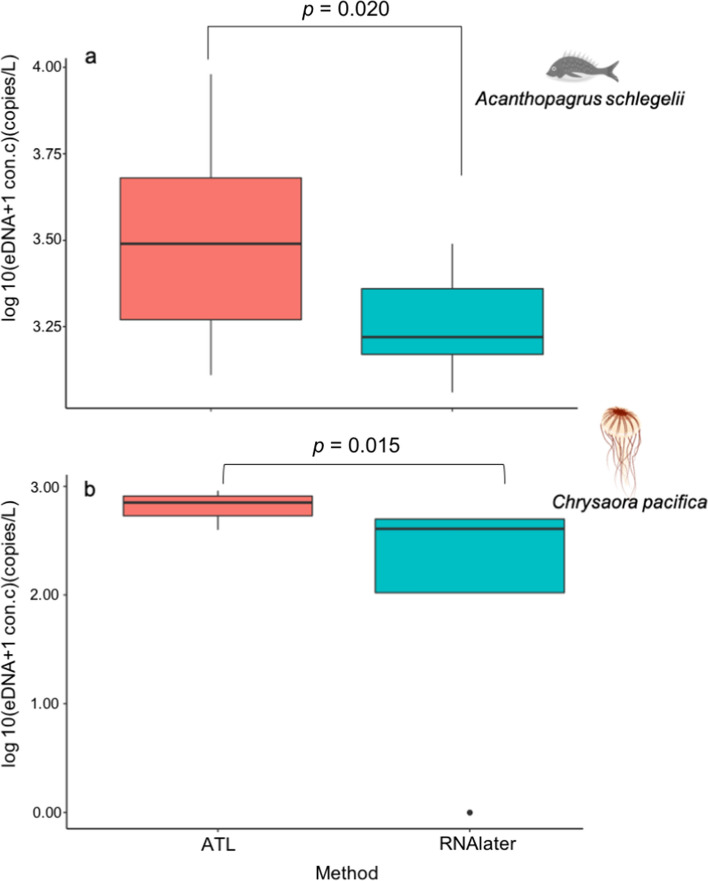

**Supplementary Information:**

The online version contains supplementary material available at 10.1007/s44211-023-00280-1.

## Introduction

In an era of severe biodiversity loss, species monitoring techniques that have minimal impact on ecosystems are essential for protecting biodiversity [1] and developing management policies [2]. Environmental DNA (eDNA) analysis has been rapidly developing as a non-destructive and non-invasive ecological monitoring technique. Environmental DNA is defined as the total DNA obtained from all organisms present in environmental media, including microbial, meiofaunal, and macrofaunal organisms, subsuming DNA from various sources such as unicellular or small multicellular organisms or tissue particles and gametes of multicellular organisms [3]. It refers to genetic substances occurring as free-floating DNA, cell fragments, feces, saliva, urine, and skin cells [4–6]. Analysis of eDNA through the collection of water samples and extraction and detection of DNA in water based on polymerase chain reaction (PCR) provides insight into the distribution and abundance of species [7–9]. This technique is widely employed in freshwater ecosystem surveys [10–12], and standardized protocols for sampling and analysis developed by the eDNA Society [13] are available. Marine ecosystems have also been investigated using eDNA analysis [14, 15]; however, few studies have been conducted on the processing of marine samples. Environmental DNA concentrations in marine samples tend to be lower than those in freshwater samples [16]; therefore, it is necessary to investigate whether protocols developed for freshwater samples can also be applied to marine samples.

To obtain reliable and precise results through eDNA analysis-based biological monitoring, it is necessary to suppress eDNA degradation in water samples [17] and improve recovery yields [18]. Some studies have demonstrated that microbial activity and a variety of abiotic factors (e.g., high temperature and acidic conditions) affect eDNA degradation [19–21]. To prevent DNA degradation during transportation, water samples are filtered in the field [22], refrigerated or frozen [23], or treated with anti-degradation reagents (e.g., Longmire’s buffer and benzalkonium chloride) following collection [24, 25]. Using marine samples, Spens et al. [26] and Miya et al. [27] proved the effectiveness of Sterivex filtration in terms of capturing DNA and showed that DNA degradation in samples can be inhibited through the addition of RNAlater [27]. Suppressing the action of DNase is critical to preserving DNA, and the effectiveness of lysis buffer in the preservation of eDNA samples has been confirmed [28]. Using Longmire’s buffer as a preservation buffer facilitates the lysis of cellular membranes, releasing intracellular components, including DNA, into the preservation buffer [29]. In most eDNA studies, DNA was extracted using a DNeasy Blood & Tissue DNA kit (Qiagen) [30–32]. Qiagen buffer-ATL (hereafter ATL) acts as a lysis buffer and has a similar effect as Longmire’s buffer in destroying cells, and thus extracts and releases DNA into the lysis buffer. Therefore, following filtration, buffer-ATL may act as a lysis buffer and preserve DNA.

In this study, two experiments were performed to determine whether preservation using either RNAlater or ATL was useful for processing marine eDNA samples. In Experiment 1, vertebrate and invertebrate species-specific quantification assays were used to determine whether different preservation methods influenced the recovery yields of eDNA from macro-organisms. In Experiment 2, a metabarcoding method was utilized to determine whether different preservation methods influenced the number of detected species and species composition.

## Materials and methods

### Experimental

#### Experiment 1: effects of different preservation on the recovery of eDNA

**Fig. 1:**
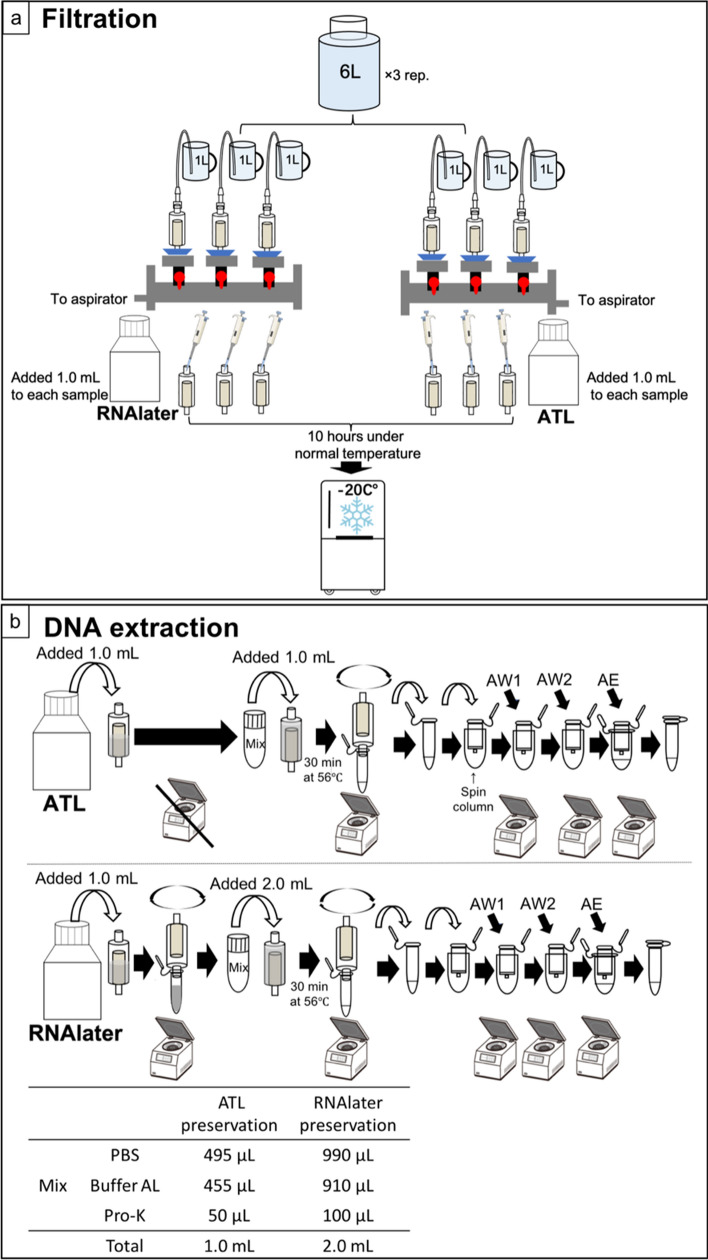
Overview of filtration and DNA extraction from Sterivex cartridges with different preservation methods. **a** Sampling site and treatments after filtering; **b** DNA extraction protocols for different preservation methods

The plastic bottles used in this experiment were pre-bleached and washed twice with the sample water prior to sample collection. Three 6 L bottles of seawater were collected from the sea-surface water at Kobe Port (34° 38′27.9" N, 135° 13′35.8" E) in Japan on June 12, 2021. All seawater samples were immediately transported to Kobe University (transit time ca. 0.5 h) and a Sterivex cartridge (0.45 μm, Millipore SVHV010RS) was used for filtering. Six 1 L bottles were filtered from each 6 L bottle (Fig. 1a). Following filtration, 1 mL RNAlater was added to each of the three samples from the inlet of the Sterivex cartridge, and an equal volume of ATL was added to each of the remaining samples (Fig. 1a). This step was repeated thrice. Additionally, 1 L of ultrapure water treated using different preservation methods was filtered and served as a filtration blank. To confirm the effect of different treatment methods on DNA recovery at room temperature, samples were first maintained at room temperature (around 25 °C) for 10 h, after which all Sterivex cartridge samples were kept at − 20 °C until DNA extraction. To prevent DNA contamination, all water collection and DNA extraction equipment were decontaminated with bleach solution (~ 0.1% sodium hypochlorite) for > 5 min to eliminate any remaining DNA.

Samples preserved with RNAlater were extracted following the method described by Wong et al*.* [33], whereas those preserved with ATL were extracted using a protocol modified from Wong et al*.* [33]. The samples preserved with RNAlater were pretreated by centrifugation during DNA extraction to remove RNAlater, and 2 mL of lysis solution (990 μL of PBS, 910 μL of Buffer AL, and 100 μL of proteinase K; Fig. 1b) was added after centrifugation. However, the samples preserved with ATL solution were not pretreated before DNA extraction. Because 1 mL of ATL solution is already present in the cartridge, we added the 1 mL lysis mix (495 μL of PBS, 455 μL of Buffer AL, and 50 μL of proteinase K; Fig. 1b) to the Sterivex cartridge. The proportion of the reagents of the lysis mix is the same as in that described by Wong et al*.* [33]. Following extraction, DNA was eluted from the DNeasy spin column with 150 μL of Buffer AE and kept at –25 °C until quantitative PCR (qPCR) analysis.

TaqMan qPCR was used to determine the eDNA concentrations of the two target species using fragments of mitochondrial cytochrome B (CytB) gene fragments of a fish species, *Acanthopagrus schlegelii,* and cytochrome c oxidase I (COI) gene fragments of a jellyfish species, *Chrysaora pacifica*. The primers and probes used were the same as those established in previous studies (Table S1) [34, 35]. Each reaction had a final volume of 20 μL included 900-nM primers, 125-nM TaqMan probe, 1 × Environmental Master Mix 2.0 (ThermoFisher Scientific), 0.1 μL AmpErase Uracil N-Glycosylase (ThermoFisher Scientific), and 2 μL of eDNA template. The qPCR conditions were as follows: 2 min at 50 °C, 10 min at 95 °C, and 55 cycles of 15 s at 95 °C, and 1 min at 60 °C. A dilution series of standards (30 to 30,000 copies/reaction) was used to obtain the calibration curves. All PCR runs, including quantification standards, extracted eDNA, filtration blank, and PCR-negative controls, were performed in triplicate, and the DNA concentration in each sample was calculated as the average of the three qPCR replicates. The DNA concentration of a replicate was considered to be zero, whenever any of the replicates produced a negative detection result [36]. The limit of detection (LOD) and the limit of quantification (LOQ) for each assay has been confirmed in previous studies [34, 35]. In each assay result, eDNA concentrations below the LOQ were regarded as “0”.

To investigate whether the different preservation methods affected the detection of eDNA concentrations of each target species, a generalized linear mixed model (GLMM) with a normal distribution was adopted. In this model, the log of eDNA concentration was set as a response variable, the type of preservation method (RNAlater or ATL) as an explanatory variable, and sampling replication as a random effect.

#### Experiment 2: effects of different preservation methods on the number of detected species and species composition by eDNA metabarcoding

The effect of different preservation methods on the number of detected species and species composition was tested by fish eDNA metabarcoding with MiFish primers. The samples collected from Experiment 1 were reused, and amplicon libraries for fish eDNA metabarcoding were prepared using MiFish-U primers [27]. The first-round PCR was conducted using 6.0 μL 2 × KAPA HiFi HotStart ReadyMix (KAPA Biosystems, Wilmington, MA, USA), 3.6 pmol of each primer, 1 μL of eDNA template, and ultrapure water with a total reaction volume of 12 μL. The thermal cycle profile was 95 °C for 3 min, followed by 40 cycles of 20 s at 98 °C, 15 s at 65 °C, and 15 s at 72 °C, and final extension of 5 min at 72 °C. For each DNA sample, four replicate PCRs were carried out. In four reaction mixtures, ultrapure water was used as a non-template negative control in place of eDNA. The products of 1st PCR were pooled into a single tube after four technical replicates and purified using the SPRI select Reagent Kit (Beckman Coulter, Brea, CA, USA) according to the manufacturer’s instructions. All samples were diluted to 0.1 ng/μL, whereas all negative controls, including filtration blanks and PCR blanks, were diluted using an average dilution ratio.

The second-round PCR was performed to add iSeq adapter and 8-bp indices to both ends of the amplicons [37]. The 2nd PCR had a 12 μL total reaction volume, including 6.0 μL of 2 × KAPA HiFi HotStart ReadyMix (KAPA Biosystems), 3.6 pmol each of forward and reverse primers, 1 μL template, and ultrapure water. The 2nd PCR’s thermal cycles profile consisted of 95 °C for 3 min; 12 cycles at 98 °C for 20 s, and 72 °C for 20 s, and the finial extension of 72 °C for 5 min. The products of the 2nd PCR were pooled into one tube to include all samples. Using the E-Gel Precast Agarose Electrophoresis System (Thermo Fisher Scientific) and E-Gel SizeSelect 2% (Thermo Fisher Scientific), the target size amplicons were extracted. An Agilent 2100 Bioanalyzer (Agilent Technologies) was subsequently used to verify whether only DNA of the target length (approximately 370 bp) was obtained. The DNA library’s concentration was adjusted to 1 nM and sequenced using the Illumina iSeq 100 System (Illumina) using 2 × 150 bp paired-end kits.

#### Sequence and statistical analyses

The raw reads obtained from iSeq sequencing were pre-processed and analyzed using USEARCH v10.0.240 [38], according to the method of Wu et al. [39]. *Homo sapiens* was detected in this analysis, and was excluded from further analysis.

In all analyses, the reads were converted to either presence or absence [40]. To estimate whether different preservation methods affected the number of detected fish species, a GLMM with a normal distribution was used. In this model, we assigned the type of preservation method (RNAlater or ATL) as an explanatory variable, the number of detected fish species as a response variable, and sampling replication as random effects.

To visualize the dissimilarity in estimated fish composition between preservation methods, non-metric multidimensional scaling (NMDS) was performed using “Jaccard methods” in 10,000 separate runs. Additionally, to compare fish composition between the two preservation methods, we performed permutational multivariate analysis of variance (PERMANOVA) and multivariate dispersion (PERMDISP) with 10,000 permutations using the “adonis” and “betadisper” functions of the vegan package, respectively. The ggplot2 package version 3.1.1 was used to construct some of the graphs [41].

## Results

**Fig. 2:**
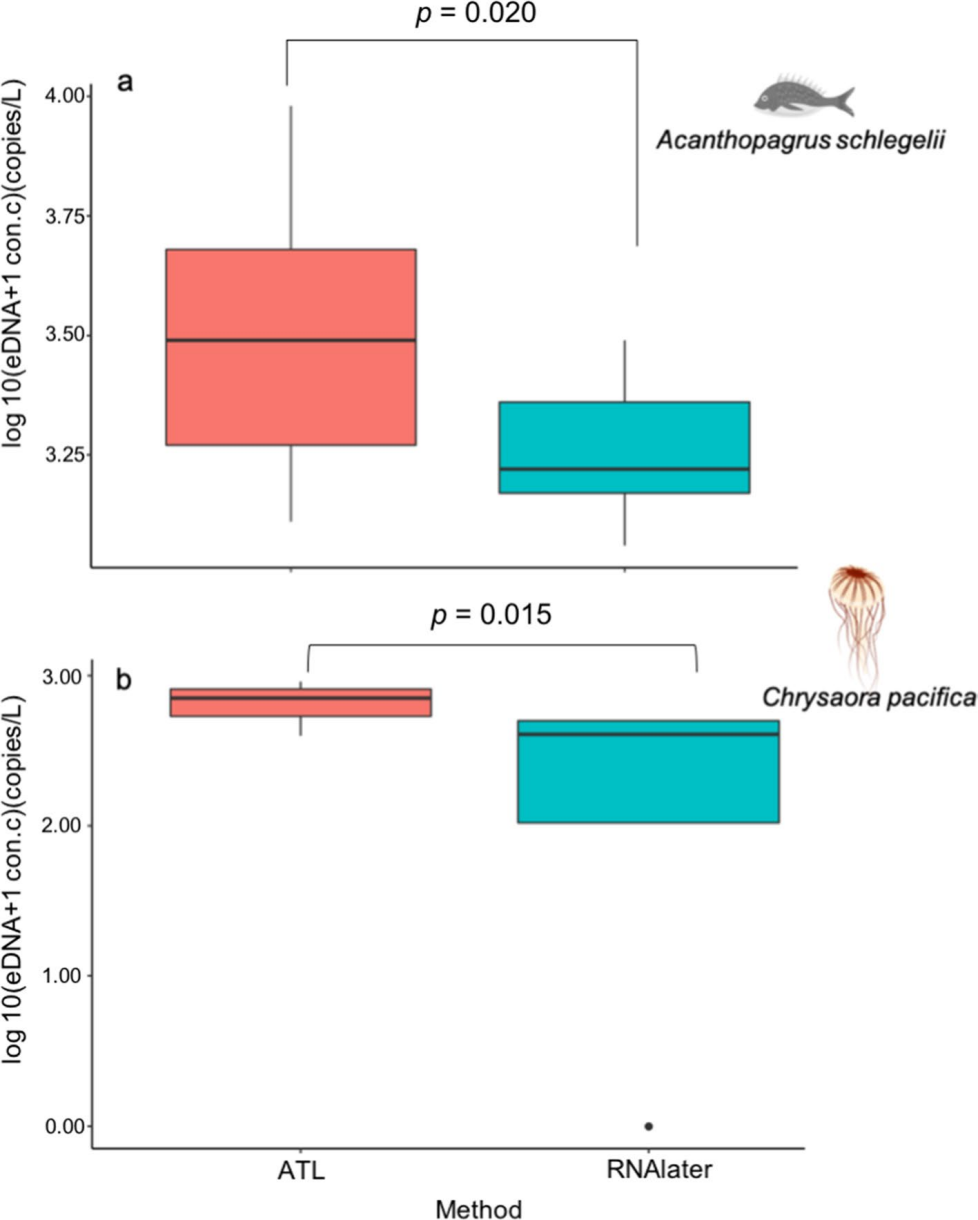
Recovered environmental DNA concentrations for each target species using different preservation methods. Results of *Acanthopagrus schlegelii* **a** and *Chrysaora pacifica* **b**

The overall PCR efficiencies and R^2^ values of the standard curves for Experiment 1 are shown in Supporting Information Table S2. The results of all PCR runs for both target species are shown in Supporting Information Table S3. Throughout the study, no amplification was observed from the filtration blank or PCR-negative controls. The concentrations of eDNA in both *A. schlegelii* and *C. pacifica* treated with ATL were higher than those in samples treated with RNAlater (GLMM, *p* = 0.020 for *A. schlegelii* [Fig. 2a] and *p* = 0.015 for *C. pacifica* [Fig. 2b]).

**Fig. 3:**
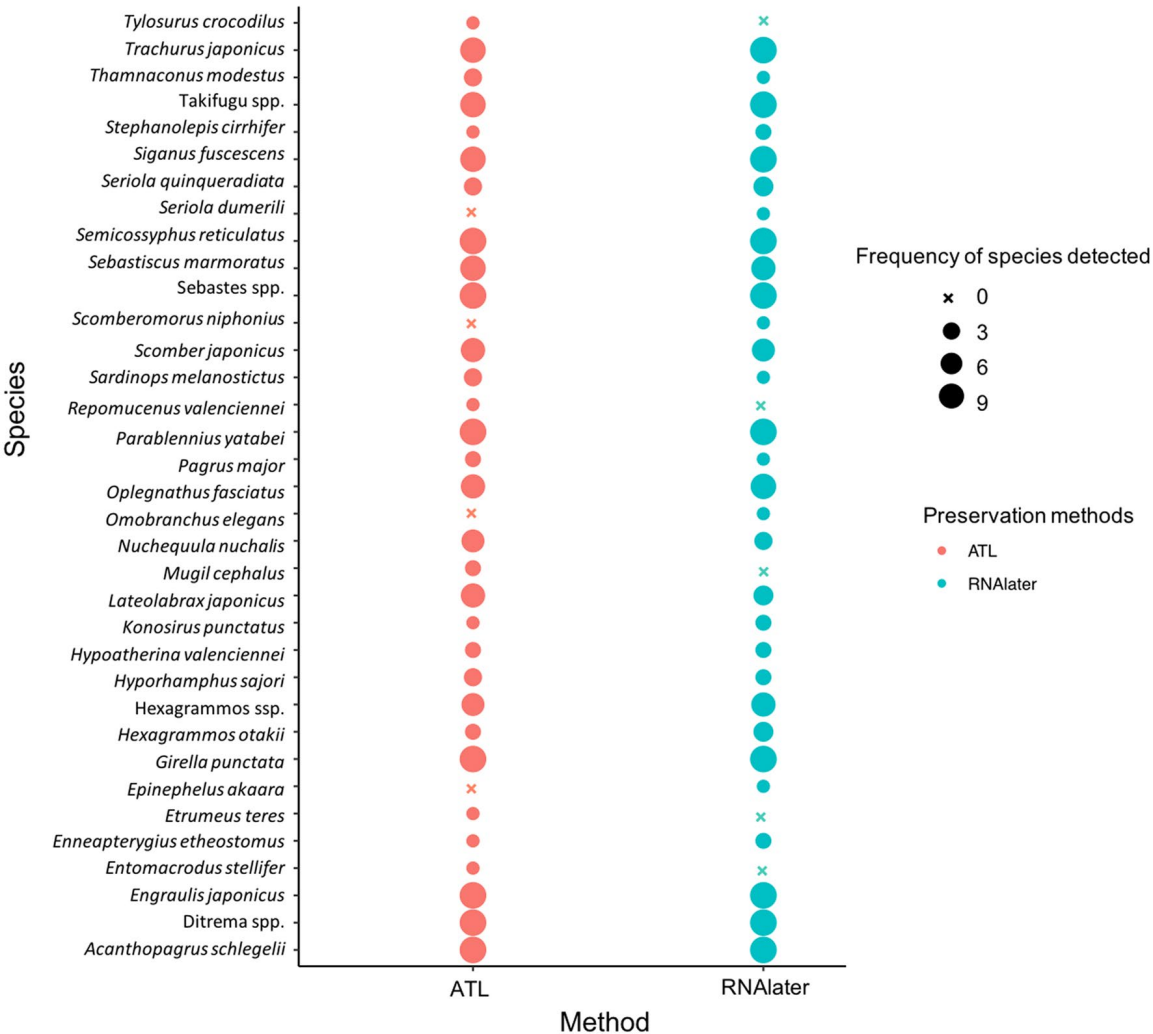
Results of detected fish species with different preservation methods. The red and blue circles indicate preservation using ATL and RNAlater, respectively. The circle size indicates the frequency of detected species in replicate sampling

**Fig. 4:**
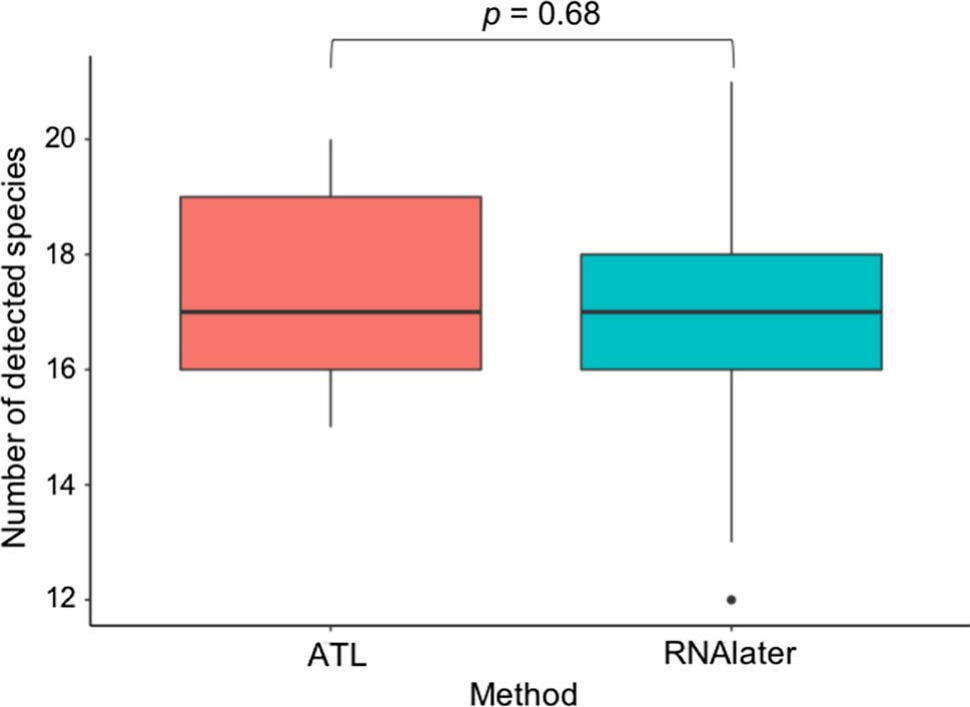
Number of fish species detected with different preservation methods by environmental DNA metabarcoding. The generalized linear mixed model (GLMM) result shows that the number of species did not differ significantly different between the different preservation methods

**Fig. 5:**
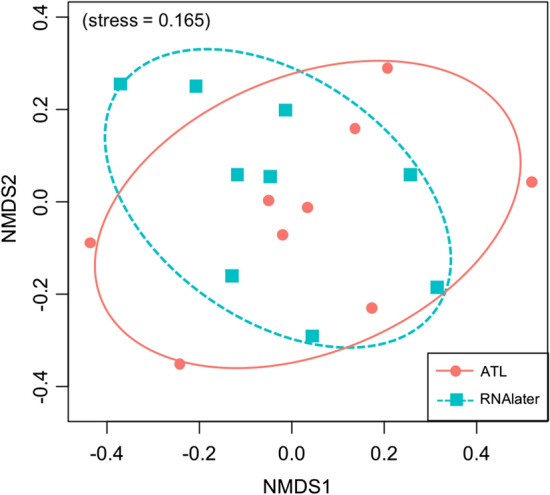
Composition of detected fish species with different preservation methods. Circles with solid represent the RNAlater-added samples (*N* = 9), circles with dotted represent the ATL-added samples (*N* = 9)

A total of 481,053 iSeq reads were obtained in Experiment 2. Following bioinformatics filtering, 283,738 reads were retained (Table S4), and fish DNA was not detected from the filtration blank or PCR-negative controls. A total of 31 and 32 fish species were detected in samples treated with RNAlater and ATL, respectively (Table S5, Fig. 3). The number of fish species detected did not differ significantly between the preservation methods (GLMM, *p* = 0.68; Fig. 4), and a non-significant difference was observed in the community composition of detected fish species (PERMANOVA, *p* = 0.48; PERMDISP, *p* = 0.95; Fig. 5).

## Discussion

In this study, the quantity and quality of macro-organismal eDNA recovered by preserving eDNA samples in ATL and RNAlater, were compared. The results of Experiment 1 showed that the eDNA concentration of the filtered samples treated with ATL was approximately twice that obtained using RNAlater. The results of Experiment 2 revealed that there was no significant difference in the number of detected fish species in the samples treated with different preservation methods, and the fish species composition was also similar. These results suggest that a greater quantity of eDNA can be recovered from samples treated with ATL, and that ATL potentially has a similar or superior preservation effect compared to RNAlater. Compared to freshwater, the eDNA concentration in seawater is relatively low and prone to false-negative results [16]. By treating seawater samples with ATL, more accurate results can be obtained by increasing the detection of low biomass species and avoiding false negatives in marine biodiversity surveys.

In this study, different preservation methods affected the eDNA recovery yield. Samples treated with RNAlater required its removal from the Sterivex cartridge by high-speed centrifugation during DNA extraction process (Fig. 1b). During centrifugation, a small quantity of cells may be expelled from the Sterivex cartridge outlet along with the liquid. Meanwhile ATL acts as a tissue lysis buffer and loosens the cell membrane (mitochondria). Samples treated with ATL were lysed by the direct addition of ProK during DNA extraction and incubated (Fig. 1a). The initial high-speed centrifugation process was omitted in this method, and the entire quantity of DNA released was recovered. This treatment method achieves increased DNA recovery. There is a potential for DNA quantity loss as the total number of centrifugations increases, and rapid centrifugation during the initial step can potentially affect the quantity of recovered DNA.

Samples processed using ATL exhibited similar preservation to those processed using RNAlater in the metabarcoding assay. The results of Experiment 2 revealed no significant difference in the number or composition of detected fish species (Figs. 3–5), suggesting that samples treated with ATL can prevent DNA degradation similar to RNAlater. Although the composition of each reagent in the Dneasy Blood and Tissue Kit is not disclosed, as ATL is a lysis solution, its DNA preservation capability is similar to that of Longmire buffer. The comparability or similarity with Longmire buffer will be investigated in future study. Higher concentrations of eDNA were observed in the samples treated with ATL (Fig. 2) in Experiment 1, and the results affected neither the number of detected species nor the composition of fish species (Figs. 3–5). It is possible that species diversity was relatively low and species eDNA concentration was relatively high in the sampled area, and this may be the reason, why, no significant difference existed between the preservation methods used. To better illustrate the effectiveness of this treatment, it is recommended that future investigations and validations be performed in areas with a higher diversity of species and/or lower biomass. Although some differences in undetected species were observed by using different preservative methods, no differences in phylogenetic or ecological characteristics were observed in the detected/undetected species. Because species detected by only one of the methods showed small numbers of reads, it may have been a stochastic fluctuation.

The analysis of eDNA is a burgeoning field. Three important considerations in eDNA research are capturing, preserving, and successfully extracting eDNA [42]. This research indicates that ATL preservation conserves DNA, and the sample processing approach used in this study may increase the DNA recovery yield. Additionally, ATL lysis buffer included as an accessory in the kit reduces costs. The findings of this study can improve the eDNA recovery yields of organisms and reduce false-negative results. Samples treated with ATL can provide highly reliable results for future biological studies.

## Supplementary Information

Below is the link to the electronic supplementary material.

Supplementary file1 (DOCX 47 KB)

## Data Availability

The authors confirm that the data supporting the findings of this study are available within the article and its supplementary materials.
