# Supplementary material for: Improvement of recovery yield of macro-organismal environmental DNA from seawater samples
Source: Anal Sci. 2023 Feb 9;39(5):713–20. doi: 10.1007/s44211-023-00280-1 (PMC10121502; doi:10.1007/s44211-023-00280-1)
Supplement: Supplementary file 1 — Supplementary file1 (DOCX 47 KB) [file 44211_2023_280_MOESM1_ESM.docx]

**Supporting Information for:**

**Improvement of recovery yield of macro-organismal environmental DNA from seawater samples**

Qianqian Wu^1*^, Toshifumi Minamoto^1^ （* Corresponding author）

^1^ Graduate School of Human Development and Environment, Kobe University: 3-11, Tsurukabuto, Nada-ku, Kobe City, Hyogo 657-8501, Japan

Table of Contents:

| Table S1 | Pages 2 |
| --- | --- |
| Table S2 | Pages 3 |
| Table S3 | Page 4 |
| Table S4 | Page 5 |
| Table S5 | Page 5 |
| Table S5 | Page 6–7 |

Table S1. Primers and probe used for species-specific qPCR.

| **Target species** | **Primers/Probe** | **Sequence (5’→3’)** | **Length** | **Reference** |
| --- | --- | --- | --- | --- |
| *Acanthopagrus schlegelii* | Asc_Cytb_F | CTGTCTGCCGTCCCCTACA | 129 bp | Takahashi et al. [34] |
|  | Asc_Cytb_R | TATGGCGGCTACGATAAAAGGA |  |  |
|  | Asc_Cytb_Pr | FAM-TCAGTTGACAACGCAACCCTAACCCG-TAMRA |  |  |
| *Chrysaora pacifica* | Cpa_COI_F | CCCAGATATGGCTTTTCCTAGA | 151 bp | Minamoto et al. [35] |
|  | Cpa_COI_R | TGAGTGAGCTTGTATAGCTGATA |  |  |
|  | Cpa_COI_Pr | FAM-TAGGATCCTCCCTAATTG-NFQ-MGB |  |  |

Table S2. R^2^ values, slopes, and Y intercepts of the calibration curves, and the PCR efficiencies for each target species.

| **Target species** | **R^2^ value** | **Slope** | **Y-Intercepts** | **PCR efficiency%** |
| --- | --- | --- | --- | --- |
| *Acanthopagrus schlegelii* | 0.994 | -3.9897 | 41.887 | 88.195 |
| *Chrysaora pacifica* | 0.990 | -3.647 | 40.419 | 93.851 |

Table S3. Quantitative real-time PCR (qPCR) for each target species with different preservation methods. Environmental DNA (eDNA) concentration was assessed using qPCR.

| **ID** | **Target species** | | | | | |
| --- | --- | --- | --- | --- | --- | --- |
|  | ***Acanthopagrus schlegelii*** | | ***Chrysaora pacifica*** | | |  |
|  | **Presence / Absence** | **eDNA concentration (copies/L)** | **Presence / Absence** | **eDNA concentration (copies/L)** |  |  |
| RNAlater1-1 | 1 | 1712.44 | 1 | 406.16 |  |  |
| RNAlater1-2 | 1 | 1632.88 | 1 | 494.73 |  |  |
| RNAlater1-3 | 1 | 2561.13 | 1 | 500.27 |  |  |
| RNAlater2-1 | 1 | 1656.20 | 1 | 104.33 |  |  |
| RNAlater2-2 | 1 | 1438.19 | 1 | 497.30 |  |  |
| RNAlater2-3 | 1 | 1156.45 | 1 | 146.30 |  |  |
| RNAlater3-1 | 1 | 1485.85 | 0 | 0.00 |  |  |
| RNAlater3-2 | 1 | 3122.36 | 1 | 411.48 |  |  |
| RNAlater3-3 | 1 | 2287.34 | 0 | 0.00 |  |  |
| ATL1-1 | 1 | 4826.56 | 1 | 634.76 |  |  |
| ATL1-2 | 1 | 7825.20 | 1 | 815.24 |  |  |
| ATL1-3 | 1 | 4471.71 | 1 | 907.46 |  |  |
| ATL2-1 | 1 | 2231.00 | 1 | 854.61 |  |  |
| ATL2-2 | 1 | 1369.64 | 1 | 393.48 |  |  |
| ATL2-3 | 1 | 1297.24 | 1 | 709.98 |  |  |
| ATL3-1 | 1 | 1866.36 | 1 | 752.84 |  |  |
| ATL3-2 | 1 | 9613.51 | 1 | 497.78 |  |  |
| ATL3-3 | 1 | 3084.19 | 1 | 538.21 |  |  |

eDNA concentrations below the limit of quantification (LOQ) are regarded as “0”.

Table S4. Detailed information on fish eDNA metabarcoding.

| **Method** | **ID** | **Pairs** | **Merged** | **Filtered reads** | **Denoise** |
| --- | --- | --- | --- | --- | --- |
| RNAlater | 1-1 | 18968 | 17686 | 11414 | 9540 |
|  | 1-2 | 28059 | 25932 | 16457 | 12949 |
|  | 1-3 | 28886 | 26976 | 17798 | 13808 |
|  | 2-1 | 26992 | 24986 | 16345 | 12889 |
|  | 2-2 | 25107 | 23270 | 14629 | 11835 |
|  | 2-3 | 31703 | 29542 | 18566 | 14020 |
|  | 3-1 | 25617 | 23588 | 14248 | 11578 |
|  | 3-2 | 25770 | 24092 | 15268 | 12478 |
|  | 3-3 | 22393 | 20944 | 13136 | 11025 |
| ATL | 1-1 | 26617 | 24842 | 15404 | 12251 |
|  | 1-2 | 29821 | 28090 | 17725 | 13285 |
|  | 1-3 | 28712 | 26740 | 16504 | 12689 |
|  | 2-1 | 30533 | 28293 | 18215 | 14050 |
|  | 2-2 | 27454 | 25865 | 16270 | 12843 |
|  | 2-3 | 22750 | 21292 | 13071 | 11102 |
|  | 3-1 | 23863 | 22345 | 14260 | 11506 |
|  | 3-2 | 29658 | 28094 | 18034 | 13389 |
|  | 3-3 | 28119 | 26540 | 16383 | 12740 |
|  | PCRNC | 31 | 23 | 11 | 0 |

Table S5. Detailed list of fish species detected from different preservation methods by eDNA metabarcoding.

| **Species name** | **RNAlater** | | | | | | | | | **ATL** | | | | | | | | |
| --- | --- | --- | --- | --- | --- | --- | --- | --- | --- | --- | --- | --- | --- | --- | --- | --- | --- | --- |
|  | **1-1** | **1-2** | **1-3** | **2-1** | **2-2** | **2-3** | **3-1** | **3-2** | **3-3** | **1-1** | **1-2** | **1-3** | **2-1** | **2-2** | **2-3** | **3-1** | **3-2** | **3-3** |
| *Acanthopagrus schlegelii* | 514 | 612 | 623 | 365 | 670 | 1437 | 224 | 746 | 603 | 788 | 1305 | 1415 | 794 | 248 | 326 | 434 | 2592 | 1346 |
| *Ditrema spp.* | 2138 | 4829 | 3618 | 3299 | 3695 | 3742 | 2998 | 3263 | 2880 | 2727 | 3914 | 2956 | 4197 | 3370 | 2684 | 1437 | 2311 | 2999 |
| *Engraulis japonicus* | 392 | 425 | 589 | 885 | 869 | 1320 | 979 | 487 | 690 | 928 | 683 | 772 | 714 | 905 | 1083 | 506 | 1130 | 913 |
| *Entomacrodus stellifer* |  | 14 |  |  |  |  | 22 |  |  |  | 28 |  |  |  |  |  |  |  |
| *Enneapterygius etheostomus* |  |  |  |  |  |  |  |  |  |  |  | 66 |  |  |  |  |  |  |
| *Etrumeus teres* |  |  | 36 |  |  |  |  |  |  |  |  |  |  |  |  |  |  |  |
| *Epinephelus akaara* |  |  |  |  |  |  |  |  |  |  |  |  |  |  | 10 |  |  |  |
| *Girella punctata* | 479 | 215 | 298 | 233 | 717 | 57 | 300 | 1554 | 153 | 350 | 266 | 258 | 689 | 717 | 121 | 583 | 238 | 442 |
| *Hexagrammos otakii* | 136 |  | 296 |  |  | 193 |  | 132 |  | 224 | 279 |  |  |  |  |  |  |  |
| Hexagrammos ssp. | 158 |  | 331 | 50 | 16 |  | 469 | 358 | 239 | 98 |  |  |  | 117 | 264 | 30 | 19 | 497 |
| *Hyporhamphus sajori* |  |  |  | 23 |  |  |  | 20 |  |  |  |  |  |  |  | 303 | 110 |  |
| *Hypoatherina valenciennei* |  | 21 |  |  |  | 32 |  |  |  | 133 | 60 | 10 |  |  |  |  |  |  |
| *Konosirus punctatus* |  |  |  |  |  |  | 125 | 103 |  |  |  |  |  |  | 15 |  |  |  |
| *Lateolabrax japonicus* | 147 | 191 | 110 |  |  |  |  | 119 |  | 126 | 340 | 803 | 130 |  | 21 | 21 | 164 |  |
| *Mugil cephalus* |  |  |  |  |  |  |  |  |  |  |  |  |  |  | 56 |  |  | 133 |
| *Nuchequula nuchalis* |  |  | 22 |  |  |  | 68 |  | 55 |  | 25 | 64 | 30 | 34 | 43 | 106 |  |  |
| *Omobranchus elegans* |  |  |  |  |  |  |  | 44 |  |  |  |  |  |  |  |  |  |  |
| *Oplegnathus fasciatus* | 188 | 105 | 17 |  | 117 | 83 | 18 | 74 | 343 | 360 | 78 | 344 | 117 | 192 |  |  | 43 | 112 |
| *Pagrus major* |  |  |  |  |  |  | 13 |  |  |  |  | 10 |  |  |  |  |  | 91 |
| *Parablennius yatabei* | 356 | 1312 | 614 | 418 | 790 | 781 | 1269 | 620 | 1120 | 746 | 1527 | 871 | 1108 | 913 | 762 | 743 | 1143 | 1096 |
| *Repomucenus valenciennei* |  |  |  |  |  |  |  |  |  |  |  |  |  |  |  | 12 |  |  |
| *Sardinops melanostictus* |  |  |  |  |  |  |  | 54 |  | 10 |  | 536 |  |  |  |  |  | 236 |
| *Scomber japonicus* | 32 | 46 |  |  |  | 74 | 156 | 64 | 59 | 121 | 12 | 20 | 102 | 41 |  |  | 75 | 47 |
| *Scomberomorus niphonius* |  | 31 |  |  |  |  |  |  |  |  |  |  |  |  |  |  |  |  |
| Sebastes spp. | 497 | 114 | 446 | 174 | 46 | 760 | 412 | 413 | 123 | 557 | 239 | 64 | 283 | 391 | 96 | 319 | 81 | 25 |
| *Sebastiscus marmoratus* | 183 | 161 | 294 |  |  | 482 | 180 | 156 | 21 | 131 |  | 87 | 15 | 198 | 114 | 329 | 39 | 283 |
| *Semicossyphus reticulatus* | 34 | 114 | 180 | 52 | 33 | 272 | 131 | 460 | 187 | 104 | 201 | 145 | 88 | 286 | 108 | 168 | 15 | 256 |
| *Seriola dumerili* |  |  |  |  |  |  |  |  | 16 |  |  |  |  |  |  |  |  |  |
| *Seriola quinqueradiata* | 12 |  | 10 |  | 104 |  |  |  | 35 |  |  | 19 | 85 |  |  |  |  | 136 |
| *Siganus fuscescens* | 281 | 336 | 2254 | 3389 | 352 | 210 | 107 | 142 | 562 | 297 | 163 |  | 776 | 1446 | 909 | 414 | 552 | 278 |
| *Stephanolepis cirrhifer* |  |  |  |  |  | 25 | 27 |  |  |  |  | 535 |  |  |  |  |  |  |
| Takifugu spp. | 570 | 1107 | 707 | 670 | 954 | 1019 | 570 | 286 | 614 | 522 | 696 |  | 1550 | 530 | 1144 | 2801 | 1564 | 478 |
| *Thamnaconus modestus* |  |  |  |  |  |  |  | 18 |  | 177 |  | 369 |  |  |  |  |  | 25 |
| *Trachurus japonicus* | 123 | 16 | 63 | 31 | 172 | 233 | 188 | 65 | 25 | 552 | 147 | 45 | 72 | 155 | 46 |  | 13 | 47 |
| *Tylosurus crocodilus* |  |  |  |  |  |  |  |  |  |  | 22 |  |  |  |  |  |  |  |
| *Homo sapiens* |  |  |  |  |  |  | 22 |  |  |  |  |  |  |  |  |  |  |  |
| Total number of eDNA reads | 6240 | 9649 | 10508 | 9589 | 8535 | 10720 | 8278 | 9178 | 7725 | 8951 | 9985 | 9389 | 10750 | 9543 | 7802 | 8206 | 10089 | 9440 |
| Total number of fish species | 17 | 17 | 18 | 12 | 13 | 16 | 20 | 21 | 17 | 19 | 18 | 20 | 16 | 15 | 17 | 15 | 16 | 19 |
